# Dynamic radiomics based on contrast-enhanced MRI for predicting microvascular invasion in hepatocellular carcinoma

**DOI:** 10.1186/s12880-024-01258-9

**Published:** 2024-04-08

**Authors:** Rui Zhang, Yao Wang, Zhi Li, Yushu Shi, Danping Yu, Qiang Huang, Feng Chen, Wenbo Xiao, Yuan Hong, Zhan Feng

**Affiliations:** 1 Department of Radiology, The First Affiliated Hospital, Zhejiang University School of Medicine, Hangzhou, China; 2 Department of Ultrasound, The Second Affiliated Hospital, Zhejiang University School of Medicine, Hangzhou, China; 3 College of Mathematical Medicine, Zhejiang Normal University School, Jinhua, China

**Keywords:** Microvascular invasion, Hepatocellular carcinoma, Magnetic resonance imaging, Dynamic radiomics, Radiomics

## Abstract

**Objective:**

To exploit the improved prediction performance based on dynamic contrast-enhanced (DCE) MRI by using dynamic radiomics for microvascular invasion (MVI) in hepatocellular carcinoma (HCC).

**Methods:**

We retrospectively included 175 and 75 HCC patients who underwent preoperative DCE-MRI from September 2019 to August 2022 in institution 1 (development cohort) and institution 2 (validation cohort), respectively. Static radiomics features were extracted from the mask, arterial, portal venous, and equilibrium phase images and used to construct dynamic features. The static, dynamic, and dynamic–static radiomics (SR, DR, and DSR) signatures were separately constructed based on the feature selection method of LASSO and classification algorithm of logistic regression. The receiver operating characteristic (ROC) curves and the area under the curve (AUC) were plotted to evaluate and compare the predictive performance of each signature.

**Results:**

In the three radiomics signatures, the DSR signature performed the best. The AUCs of the SR, DR, and DSR signatures in the training set were 0.750, 0.751 and 0.805, respectively, while in the external validation set, the corresponding AUCs were 0.706, 0756 and 0.777. The DSR signature showed significant improvement over the SR signature in predicting MVI status (training cohort: *P* = 0.019; validation cohort: *P* = 0.044). After external validation, the AUC value of the SR signature decreased from 0.750 to 0.706, while the AUC value of the DR signature did not show a decline (AUCs: 0.756 vs. 0.751).

**Conclusions:**

The dynamic radiomics had an improved effect on the MVI prediction in HCC, compared with the static DCE MRI-based radiomics models.

## Background

Hepatocellular carcinoma (HCC) is the fourth most deadly cancer worldwide [1]. Liver resection and transplantation are the most effective curative treatment methods, although the postoperative recurrence rate remains high [2]. Microvascular invasion (MVI) is an independent risk factor of early recurrence for HCC [3]. Therefore, preoperative prediction of MVI may be essential for treatment strategies [4].

As MVI is only reliably diagnosed by histopathology, it is challenging to achieve a non-invasive preoperative diagnosis. Traditional radiological characteristics, such as intratumoral arteries, have shown to be conducive to MVI diagnosis [5] but be inferior to radiomics signatures [6–9]. However, previous radiomics studies were almost entirely based on static radiomics features, ignoring the changes in features over time. Recently, Qu et al. proposed a feature extraction method called dynamic radiomics [10], which captured the feature change pattern in the time dimension using three types of featuring methods, including integrated features, discrete features, and parameter fitting features. These dynamic features revealed the tumor heterogeneity, metabolic changes, and tumor angiogenesis information. The occurrence of MVI in patients with HCC is accompanied by changes in blood supply and metabolism in the tumor microenvironment [11–13]. Thus, it is reasonable to investigate whether dynamic radiomics on multi-phase dynamic contrast-enhanced (DCE) magnetic resonance imaging (MRI) may allow more effective MVI prediction.

In this study, we exploited the improved prediction performance based on multi-phase DCE-MRI using dynamic radiomics features for the preoperative MVI in HCC.

## Materials and methods

### Patients

**Fig. 1:**
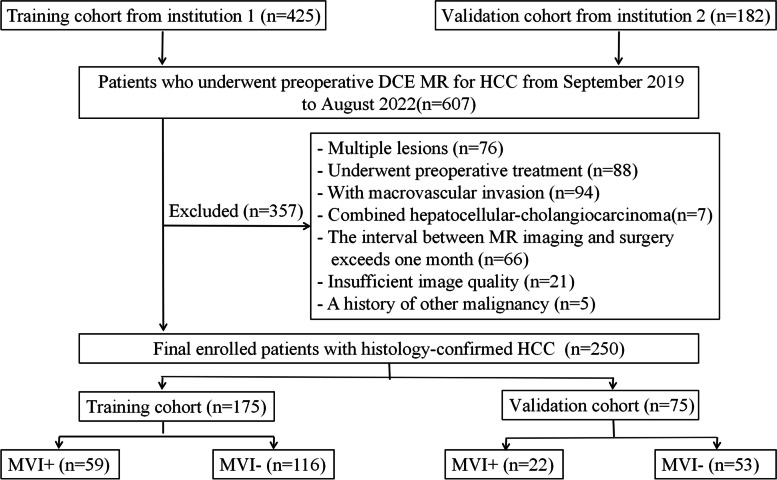
Flowchart of the study patients. DCE MRI dynamic contrast-enhanced MRI, HCC hepatocellular carcinoma, MVI microvascular invasion

This retrospective study was approved by the institutional review board, and the requirement for informed consent was waived. All patients with HCC undergoing three-phase preoperative DCE-MRI between September 2019 and August 2022 were enrolled. The inclusion criteria were as follows: (1) pathologically confirmed HCC; (2) singular tumors, with or without satellite nodules—defined as lesions with a diameter ≤ 2 cm and a distance ≤ 2 cm from the main tumor; (3) DCE-MRI was performed within 1 month before surgery; (4) no prior cancer therapy, including transarterial chemoembolization and radiofrequency ablation; and (5) no macrovascular invasion shown on MRI. The exclusion criteria were as follows: (1) special types of liver cancer such as double phenotype liver cancer; (2) poor MRI image quality; (3) recurrent HCC; and (4) concurrence of other malignancies. Figure 1 shows the patient recruitment process.

### Laboratory examination and histopathology

Preoperative laboratory indexes (Table 1) included serum α-fetoprotein (AFP), carcinoembryonic antigen, carbohydrate antigen 199, carbohydrate antigen 125, ferritin, hepatitis B virus, total bilirubin, direct bilirubin, albumin, and gamma-glutamyl transferase (GGT). The Barcelona Clinic Liver Cancer (BCLC) staging system was also incorporated.

**Table 1:** Clinicoradiological characteristics in training and validation cohorts

Variables	Training cohort (*n* = 175)	Validation cohort (*n* = 75)	*P*-value
MVI (negative/positive)	116/59	53/22	0.498
Age, mean ± SD, years	58.35 ± 11.03	58.25 ± 8.85	0.939
Sex (male/female)	154/21	61/14	0.164
BCLC (0/A)	15/160	11/64	0.148
Hepatitis B virus (present/absent)	153/22	65/10	0.869
Cirrhosis (present/absent)	137/38	54/21	0.283
Albumin (≤ 65/ > 65 g/L)	13/162	8/67	0.398
GGT (≤ 60/ > 60 U/L)	95/80	37/38	0.472
AFP (≤ 20/ > 20 ng/ml)	82/93	44/31	0.087
Tumor size,mean ± SD,cm	4.47 ± 2.40	4.34 ± 2.4	0.688
Gross type (nodular/ non-nodular)	43/132	23/52	0.316
Arterial peritumoral enhancement (present/absent)	47/128	14/61	0.167
Arterial rim enhancement (present/absent)	24/151	17/58	0.080
Wash out (present/absent)	143/32	62/13	0.857
Peritumoral hypointensity (present/absent)	31/144	13/62	0.942
Radiological capsule (absence or incomplete/complete)	133/42	61/14	0.354
Internal arteries (present/absent)	81/94	28/47	0.191

*Abbreviations:* *AFP *Serum α-fetoprotein, *BCLC *Barcelona Clinic Liver Cancer, *GGT* Gamma-glutamyltransferase, *MVI* Microvascular invasion

*p* value < 0.05: a significant difference between the training and validation cohorts, which was enrolled in the t-test, or chi-square test

HCC pathological specimens were collected following the 7-point baseline sampling protocol. Histopathological characteristics, including MVI status and liver fibrosis grade based on the Scheuer scoring system, were double-blindly determined by two pathologists with more than 10 years of experience. MVI means the detection of cancer cell nests in the vascular lumen lined with endothelial cells under a microscope, it is mainly observed in the branches of the portal vein.

### DCE-MRI protocol

All MR examinations were performed with a 3.0 T MRI system (Signa HDXT, GE Medical Systems, Milwaukee, WI, USA) with intravenous bolus injection of 0.1 mmol/kg gadopentetate dimeglumine (Magnevist®, Bayer Schering Pharma, Berlin, Germany) in both institutions. Scans in a three-dimensional fast-spoiled gradient-recalled echo sequence (liver acceleration volume acquisition, LAVA) at the arterial, portal vein, and equilibrium phases were obtained with 20—35 s, 60—90 s, and 160—180 s delays, respectively. The scanning parameters for institution 1 were as follows: repetition time, 3.2 ms; echo time, 1.5 ms; reversal angle, 10°; field of view, 380 × 304 mm; thickness, 2 mm. While the scanning parameters for institution 2 were as follows: repetition time, 3.5 ms; echo time, 1.6 ms; reversal angle, 13°; field of view, 380 × 380 mm; thickness, 2 mm.

### Qualitative radiographic descriptors

Image analysis was performed double-blindly by two radiologists with 10 and 15 years of experience in liver MRI diagnosis. The following eight imaging characteristics were assessed: (a) tumor size; (b) tumor gross type—nodular or non-nodular [14]; (c) rim enhancement in the arterial phase [15]; (d) arterial peritumoral parenchymal enhancement [16]; (e) washout [17]; (f) peritumoral hypointensity in the later phase [18]; (g) radiological capsule [19]; and (h) intratumoral artery [5].

### Tumor segmentation and static radiomics feature extraction

The whole tumor was manually depicted along with the lesion outline on each axial slice of each MRI phase by a radiologist with 10 years of experience ( reader 1) using ITK-SNAP (http://www.nitrc.org/projects/itk-snap/). Additionally, to evaluate the intra-observer reproducibility and inter-observer reliability of feature extraction, images of 40 patients randomly selected from the training cohort after 3 months were resegmented independently by two radiologist with 10 (reader 1) and 7 (reader 2) years of experience, respectively. The intra-/inter-class correlation coefficients (ICCs) were used to test the consistency between intra-observer and inter-observer ROIs. For images with different resolutions, all voxel sizes of all images were resampled with the same size of 1 × 1 × 1 mm^3^. The image gray-scale values were normalized. The normalization procedure was based on the following mathematical formula:

1 $$x^{'}= (x - \mu ) /\sigma$$

where μ is the mean image density value, σ is the standard deviation of image density. The gray values of the images were normalized to 1–64, as recommended by Orlhac F et al. [20]. Quantitative radiomics parameters were calculated using MATLAB software. A total of 484 radiomics features were obtained and classified into three categories, including 7 intensity features, 53 texture features, and 424 wavelet features.

### Dynamic radiomics feature construction

Dynamic radiomics features were constructed based on the static features change of the same imaging examination at different phases or different imaging examinations, which can be expressed as Eq. 2:

2 $$\phi(\Psi(x(t1)),\Psi(x(t2)),\dots,\Psi(x(tk)))$$

where *Ф*(^.^) transforms *R*^*k*^ to *R*^*d*^. *k* is the phase number of the image, and *d* is the number of extractable dynamic features. The following three types of dynamic features were constructed to reflect the changes in static features in different phases:

1. Integrated features.

The integrated features mainly describe the pattern of feature changes with respect to time. Three types of integrated features were studied, including the mean, variance, and coefficient of variation.

2. Discrete features.

The discrete features mainly describe the pattern of feature changes between two consecutive time points (defined as a segment) and involve two calculation methods: relative change rate (RCR) and relative average change rate (RACR), which are calculated as in Eqs. 3 and 4

3 $$RCR(\Psi (x(t))) = |\Psi (x(tj)) - \Psi (x(ti))| / \Psi (x(ti)), 1 \le j \le i \le k$$

4 $$RACR(\Psi (x(t))) = |\Psi (x(tj)) - \Psi (x(ti))| / \Psi (x(\dot{t})), 1 \le j \le i \le k$$

In this study, there were four scanning time points, corresponding to three segments: plain–arterial phase, arterial–portal vein phase, and portal vein–equilibrium phase. Six types of discrete features were obtained.

3. Parameter fitting features.

Linear, quadratic, and exponential lines were fitted to the feature–time relationships. Parameters of the three fitting methods, with the maximum curvatures of the quadratic and exponential fittings, were recorded as dynamic features for the corresponding static feature.

The linear fitting equation is expressed as Eq. 5, and* k* and *d* were extracted as dynamic features.

5 $$feature = k \times t + d$$

The quadratic fitting equation is expressed as Eq. 6, and *a*, *b*, and *c* were extracted as dynamic features.

6 $$feature=a\times t^2+b\times t+c$$

The curvature of the quadratic function is expressed as Eq. 7. The time (*T*_maxQK_) corresponding to the maximum curvature (max*Q*K) and the curvature (*QK*_max_feature_) corresponding to the maximum feature value were solved for and recorded as two dynamic features.

7 $$QK=\left|2a\right|/{(1+\left(2a\times t+b\right)^2)}^{3/2}$$

The exponential fitting equation is expressed as Eq. 8, and *α* and *β* were obtained as dynamic features.

8 $$feature=\alpha\times e^t+\beta$$

The curvature of the exponential function is expressed as Eq. 9. The time (*T*_maxEK_) corresponding to the maximum curvature (maxEK) and the curvature (*EK*_max_feature_) corresponding to the maximum feature value were solved for and recorded as two dynamic features.

9 $$EK=\left|a\times {e}^{t}\right|/{\left(1+a\times {e}^{2t}\right)}^{3/2}$$

A total of 20 types of dynamic radiomics features were obtained. For each patient, the number of static radiomics features was 484, and the number of dynamic radiomics features was 20 × 484.

### Radiomics signature construction

The variability between the two radiologists’ tumor contours was estimated using ICCs. Stable features with ICCs > 0.8 were used for analysis. The segmentation data set of all images after the first segmentation by reader 1 was adopted. All features were standardized into a normal distribution with z-scores to eliminate index dimension differences of the data. Model training procedure was fed into a repetitive (5 runs) fivefold cross- validation approach using the training set. For the construction of static radiomics (SR) signature, the final feature set of each ROI comprised a total of 1936 static radiomics features, encompassing four phases. Firstly, F-test was used to screen for features associated with MVI, the least absolute shrinkage and selection operator (LASSO) was then used to screen the most informative image features. Finally, logistic regression analysis was utilized to integrate the selected features to establish the SR signature. For the construction of dynamic radiomics (DR) signature, a total of 9680 dynamic radiomics features were obtained per patient. F-test and LASSO were used o screen the most informative image features. A logistic regression classifier was then used for the DR signature establishment. For the construction of dynamic-static radiomics ( DSR) signature, firstly, the optimal features selected for both the SR and DR signatures were combined. Then, these features were screened once more using F-test and LASSO to derive the DSR signature through logistic regression. Classification accuracy, the area under the receiver operating characteristic (ROC) curve (AUC), sensitivity and specificity were used to evaluate the predictive performance of each radiomics signature. ROC curves and precision-recall curves were plotted to evaluate and compare the predictive performance of each signature.

### Statistical analysis

The Dr. Wise Multimodal Research Platform (https://keyan.deepwise.com, V1.6.2; Beijing Deepwise & League of PHD Technology Co., Ltd, Beijing, China) was used for radiomics feature selection and modeling. Clinical data were analyzed using descriptive statistics, numerical data were analyzed using the t-test, and categorical data were analyzed using the chi-square test. Statistical significance was assigned when two-sided *p*-values were < 0.05.

## Results

### Clinicoradiological characteristics

In this retrospective study, 175 patients in institution 1 were used as the training set, including 59 MVI-positive and 116 MVI-negative patients. 75 patients in institution 2 were used as the external validation set, including 22 MVI-positive and 53 MVI-negative patients. A total of 250 patients were included in the study (215 males and 35 females; average age: 58.34 ± 10.41 years; range: 31–38 years). The clinical-radiological characteristics of the training and validation cohorts are listed in Table 1. There were no significant differences in clinical-radiological characteristics between the two cohorts.

### Performance of the static, dynamic, and dynamic–static radiomics signatures

**Fig. 2:**
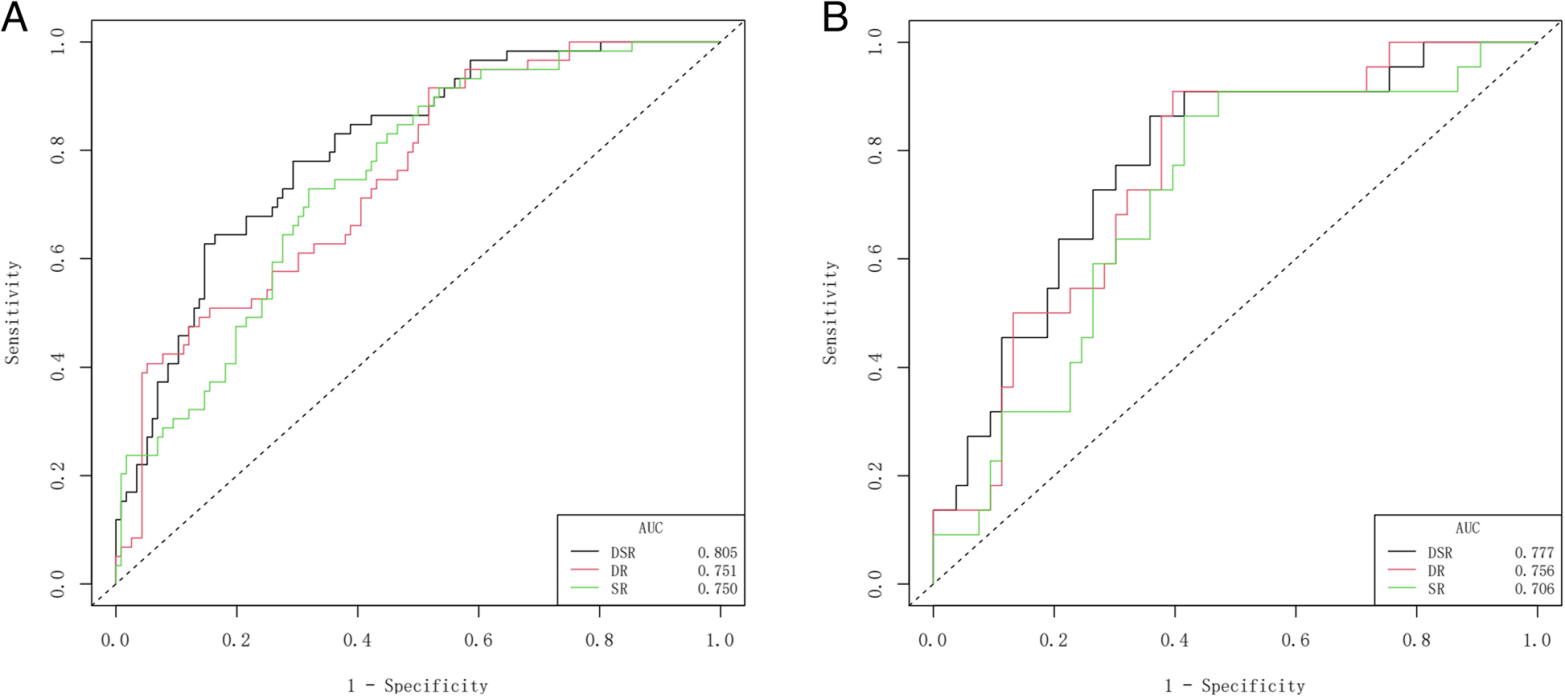
ROC curves of different models for predicting MVI in the training (**A**) and validation (**B**) cohort. DR dynamic radiomics, DSR dynamic-static radiomics, ROC receiver operating characteristic, MVI microvascular invasion, SR static radiomics

**Fig. 3:**
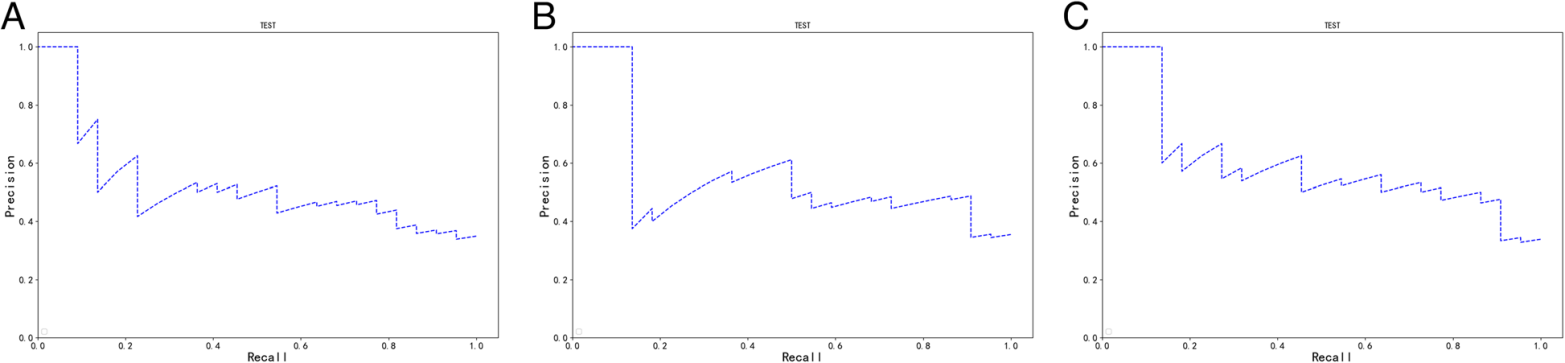
Precision-recall curves of static (**A**), dynamic (**B**) and dynamic-static (**C**) radiomics signatures or predicting MVI in the validation cohort. MVI microvascular invasion

For patients with HCC, the MVI prediction performance of the SR, DR, and DSR signatures based on three-phase DCE-MRI in the training and validation cohorts is shown in Table 2 and Figs. 2 and 3. In the three radiomics signatures, the DSR signature performed the best. The AUCs of the SR, DR, and DSR signatures in the training set were 0.75, 0.751 and 0.805, respectively, while in the external validation set, the corresponding AUCs were 0.706, 0756 and 0.777. The DSR signature showed significant improvement over the SR signature in predicting MVI status (training cohort, *P* = 0.019; validation cohort, *P* = 0.044). After external validation, the AUC value of the SR signature decreased from 0.750 to 0.706, while the AUC value of the DR signature did not show a decline (AUCs: 0.756 vs. 0.751).

**Table 2:** Performance of the static, dynamic, and dynamic–static radiomics signatures

Signatures	Training set (*n* = 175)				Validation set (*n* = 75)			
	AUC (95%CI)	Accuracy	Sensitivity	Specificity	AUC (95%CI)	Accuracy	Sensitivity	Specificity
SR	0.750(0.677–0.822)	0.640	0.746	0.586	0.706 (0.579–0.833)	0.693	0.864	0.585
DR	0.751(0.678–0.825)	0.623	0.661	0.603	0.756 (0.641–0.870)	0.667	0.727	0.642
DSR	0.805(0.739–0.871)	0.714	0.780	0.681	0.777 (0.663–0.891)	0.720	0.773	0.698
*P*-value	*P*1 = 0.962, *P*2 = 0.019				*P*1 = 0.302, *P*2 = 0.044			

*Abbreviations*: *SR* Static radiomics, *DR* Dynamic radiomics, *DSR* Dynamic-static radiomics, *AUC* Area under the curve, *CI* Confidence interval

*P*1, AUC comparison between SR and DR; *P*2, AUC comparison between SR and DSR

**Fig. 4:**
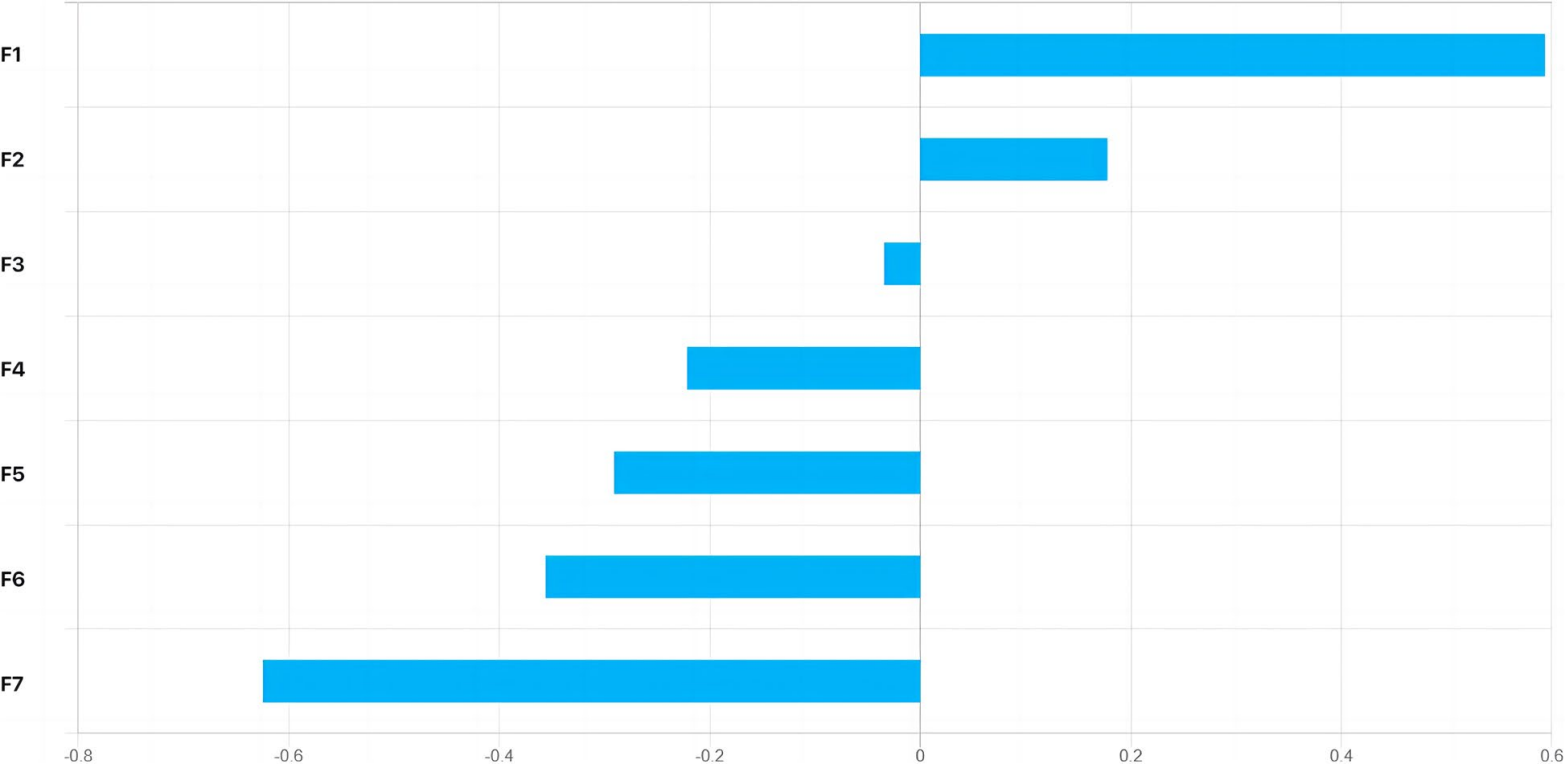
Plot of feature importance for the static radiomics signatures in HCC. F1: Static feature—Skewness—arterial phase, F2: Static feature—Small zone emphasis_wavelet.LLH—arterial phase, F3: Static feature—Long run low gray-level emphasis_wavelet.LLH—arterial phase, F4: Static feature—Variance—equilibrium phase, F5: Static feature—Variance—arterial phase, F6: Static feature—Maximum probability_wavelet.LHL—portal venous phase, F7: Static feature—Long run emphasis_wavelet.LLH—arterial phase

**Fig. 5:**
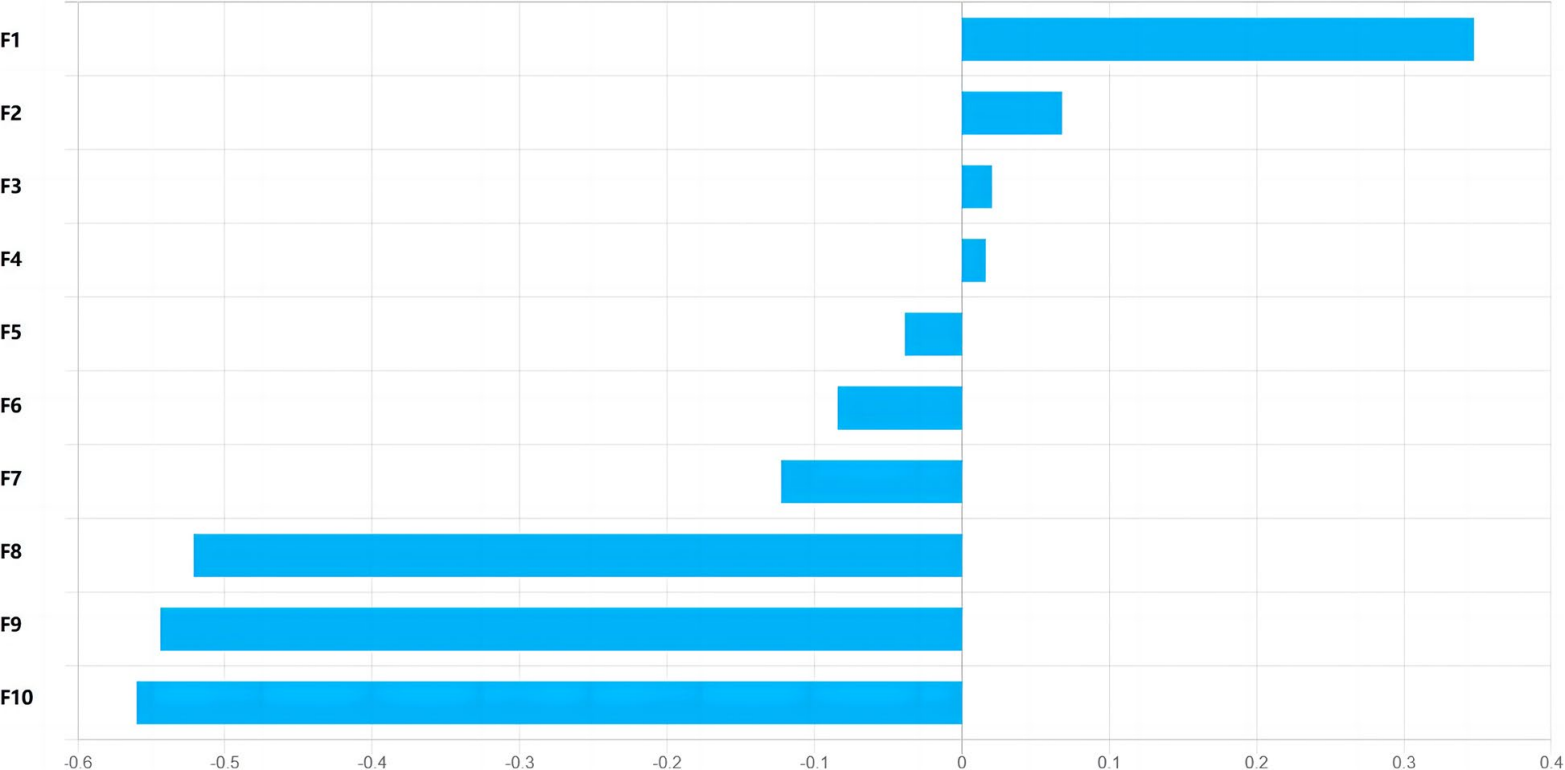
Plot of feature importance for the dynamic radiomics signatures in HCC. F1: Discrete feature_arterial–portal vein phase (RACR)—Contrast_wavelet.LLL, F2: Exponential fitting feature *α*—Cluster prominence_wavelet.LLH, F3: Exponential fitting feature *α*—Correlation2_wavelet.LLH, F4: Quadratic fitting feature *c*—Correlation1_wavelet.HLL, F5: Discrete feature_plain–arterial phase (RACR)—Autocorrelation_wavelet.LLL, F6: Integrated feature_variance—Cluster prominence_wavelet.LLH, F7: Integrated feature_mean—Cluster prominence_wavelet.LLH, F8: Integrated feature_mean—Variance, F9: Quadratic fitting feature *c*—Long run low gray-level emphasis_wavelet.HLL, F10: Integrated feature_ coefficient of variation—Gray-level variance_wavelet.HLH

**Fig. 6:**
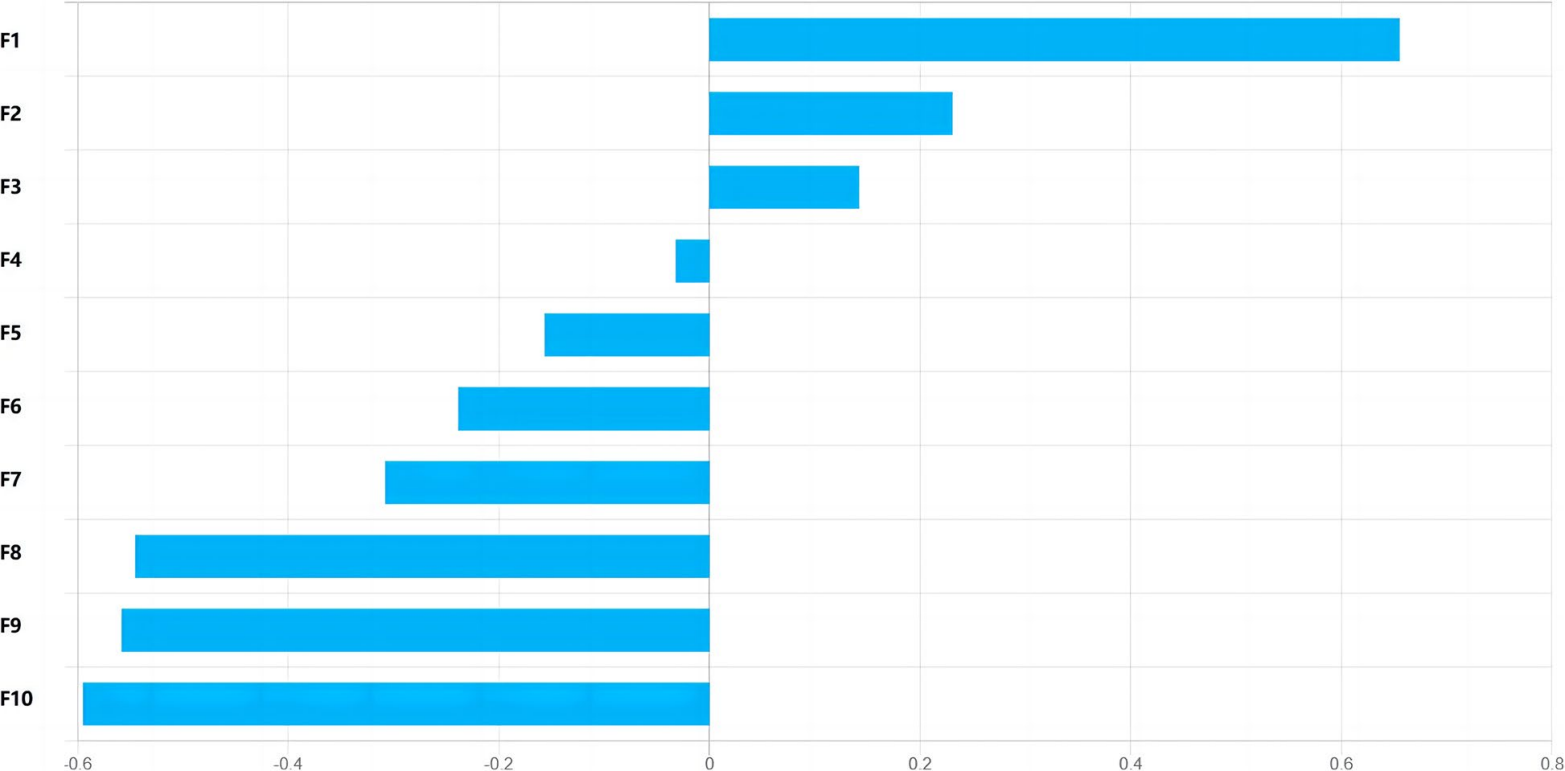
Plot of feature importance for the dynamic-static radiomics signatures in HCC. F1: Static feature—Skewness—arterial phase, F2: Static feature—Small zone emphasis_wavelet.LLH—arterial phase, F3: Discrete feature_arterial–portal vein phase (RACR)—Contrast_wavelet.LLL, F4: Static feature—Variance—arterial phase, F5: Static feature—Variance—equilibrium phase, F6: Integrated feature_mean—Variance, F7: Static feature—Maximum probability_wavelet.LHL—portal venous phase, F8: Quadratic fitting feature *c*—Long run low gray-level emphasis_wavelet.HLL, F9: Static feature—Long run emphasis_wavelet.LLH—arterial phase, F10: Integrated feature_ coefficient of variation—Gray-level variance_wavelet.HLH

The optimal contributing feature sets for the SR, DR, and DSR signatures in HCC are given in Figs. 4, 5 and 6. The 7 most valuable static radiomics features were selected to construct the SR signature, including 5 features of arterial phase, 1 features of portal venous phase, and 1 features of equilibrium phase (Fig. 4). The 10 most valuable features were used to construct the DR signature, including 4 integrated features, 2 discrete feature, and 4 parameter fitting features (Fig. 5). The 10 most significant features were selected to construct the DSR signature based on three-phase DCE-MRI. These included 6 static radiomics features and 4 dynamic radiomics features (Fig. 6).

## Discussion

In this study, we used a new dynamic radiomics method based on DCE-MR images for predicting MVI in HCC. The dynamic features described the changes of static features in different phases and revealed more comprehensive imaging information. The combined dynamic–static radiomics model showed an improvement in the prediction of MVI in HCC, which helped with patient stratification and treatment personalization.

Preoperative prediction of MVI in patients with HCC is of great significance for clinical treatment decisions. Recently, many studies have confirmed that radiomics could be an accurate and effective tool for MVI prediction in HCC patients [21, 22], which holds promise for the non-invasive prediction and personalized treatment. However, previous radiomics studies have only focused on static radiomics and have ignored the change pattern of the static features over time. Recently, Qu et al. proposed a dynamic radiomics feature construction method to describe the change pattern of static features of the same or different imaging examinations over time [10]. The method was confirmed to be superior to static radiomics in the diagnosis and prognosis prediction of some cancers [23, 24]. The occurrence of MVI in patients with HCC is accompanied by changes in blood supply in the tumor microenvironment [12, 13]. DCE-MRI [25] scans at different phases after intravenous injection of paramagnetic contrast agent to show the process of contrast agent perfusion and clearance, thus reflecting the microcirculation status of the tumor. DCE-MRI has been used in the preoperative MVI assessment of HCC [13]. Dynamic radiomics analysis of DCE-MRI images could identify the microscopic structure of lesions in a quantitative approach and capture more hidden information. To the best of our knowledge, our study is the first to use dynamic radiomics method based on DCE-MR images to predict MVI in HCC. In this study, the dynamic–static radiomics signature showed the best predictive power among the static, dynamic, and dynamic–static radiomics signatures. In terms of the reasons for this observation, compared to static radiomics, dynamic radiomics had the following advantages: (1) dynamic features reflected the changes in static features over time and revealed blood supply and metabolic information of the tumor, which led to better modeling; and (2) the dynamic features contained the relative changes in the static features and reduced the effect of inter-image and inter-patient variability.

MVI was defined as a nest of malignant cells in vessels only visible by microscopy [26]. The vascular lumen was mainly composed of portal vein branches adjacent to cancer. The presence of MVI theoretically leads to perfusion behavior changes within the lesion: blockage of small branches of portal vein can decrease portal vein blood flow, and in return, lead to excessive arterial blood flow perfusion; MVI can cause vascular reconstruction, reduce the adhesion of vascular endothelial cells, and thus reduce portal vein resistance [27]. These will entail changes in blood flow perfusion within the lesion. Zhang L et al. [12] used perfusion parameters from conventional three-phase CT scans to help predict MVI preoperatively, showing that portal vein blood supply perfusion (PVP), arterial enhancement fraction (AEF), hepatic artery perfusion Index (HPI), and their related parameters had certain value in predicting MVI, and the combination of PVP, AEF and HPI had the highest diagnostic efficacy, with an AUC value of 0.741. In our study, the DSR signature exhibited better performance, with an AUC value of 0.805 in the training set and 0.777 in the external validation set. Compared to perfusion parameters, dynamic radiomics analysis could identify the microscopic structure of lesions in a quantitative approach and capture more hidden information.

In this study, as shown in Figs. 4, 5 and 6, the skewness and variance adopted by the signatures are both histogram features, describing the statistical distribution characteristics of voxel intensity within the ROI. Among the texture features adopted by the signatures, the maximum probability, cluster prominence, correlation 1, correlation 2, and autocorrelation belong to Gray-level co-occurrence matrix (GLCM), GLCM describes the distribution of voxel intensities along specific directions and distances within the ROI. Long run low gray-level emphasis, long run emphasis, and gray-level variance all belong to Gray-level run-length matrix (GLRLM), GLRLM describes the distribution patterns of runs with the same gray-level intensity arranged along specific directions within the ROI. Small zone emphasis belongs to Gray-level size zone matrix (GLSZM), GLSZM describes the distribution patterns of regions with the same gray-level intensity within the ROI. Contrast belongs to Neighborhood gray-tone difference matrix (NGTDM), NGTDM describes the difference in gray-levels between any central voxel and its surrounding neighborhood voxels within the ROI. Texture features based on wavelet transform belong to higher-order features, which extract details of the image by using different frequency filters along the x, y, and z axes in space. If HLH represents the image being filtered through the high-pass filter in the x-direction, the low-pass filter in the y-direction, and the high-pass filter in the z-direction, then the wavelet transform methods adopted by the three signatures include: LLH, LLL, LHL, HLL, and HLH.

This study has several limitations. First, there are variations in scanning parameters between the two institutions. To address this issue, pre-processing operations were conducted on the images to mitigate the impact of these variations in image acquisition parameters. Although this is considered a limitation, it demonstrates the reproducibility and generalizability of our study. Second, hepatobiliary contrast agents were not used. The imaging performance of hepatobiliary contrast agent is not ideal in the arterial phase and may lead to poor feature extraction [28, 29]. Besides, hepatobiliary contrast agent is not recommended for diagnosing HCC by the American Association for the Study of Liver Diseases (AASLD) [30]. Third, the method of extracting dynamic features still needs to be further optimized. However, despite these problems, we still believe that dynamic radiomics has great potential in disease diagnosis and prognosis assessment.

## Conclusions

In summary, compared to static radiomics, dynamic radiomics approach can provide added value for MVI prediction in HCC. The application of the combined dynamic-static radiomics model to predict the MVI status of HCC has strong clinical significance and broad development prospects.

## Data Availability

The datasets used and/or analysed during the current study are available from the corresponding author on reasonable request.

## Abbreviations

AFP: Alpha-fetoprotein
AUC: Area under the curve
DCE MRI: Dynamic contrast-enhanced magnetic resonance imaging
DR: Dynamic radiomics
DSR: Dynamic-static radiomics
HCC: Hepatocellular carcinoma
ROC: Receiver operating characteristic
MVI: Microvascular invasion
SR: Static radiomics
